# Genetic structure in insular and mainland populations of house sparrows (*Passer domesticus*) and their hemosporidian parasites

**DOI:** 10.1002/ece3.1452

**Published:** 2015-03-23

**Authors:** Coraline Bichet, Yoshan Moodley, Dustin J Penn, Gabriele Sorci, Stéphane Garnier

**Affiliations:** 1 Biogéosciences, UMR CNRS 6282, Université de Bourgogne6 Boulevard Gabriel, 21000, Dijon, France; 2 Laboratoire LBBE, UMR CNRS 5558, Université Claude Bernard Lyon 1bâtiment Mendel, 43 boulevard du 11 novembre 1918, 69622, Villeurbanne Cedex, France; 3 Department of Zoology, University of VendaPrivate Bag X5050, Thohoyandou, 0950, South Africa; 4 Department of Integrative Biology and Evolution, Konrad-Lorenz-Institute of Ethology, University of Veterinarian Medicine ViennaSavoyenstr. 1a, A-1160, Vienna, Austria

**Keywords:** Genetic differentiation, genetic variability, haemosporidian parasites, insularity, major histocompatibility complex, microsatellites, *Passer domesticus*

## Abstract

Small and isolated populations usually exhibit low levels of genetic variability, and thus, they are expected to have a lower capacity to adapt to changes in environmental conditions, such as exposure to pathogens and parasites. Comparing the genetic variability of selectively neutral versus functional loci allows one to assess the evolutionary history of populations and their future evolutionary potential. The genes of the major histocompatibility complex (MHC) control immune recognition of parasites, and their unusually high diversity is genes which is likely driven by parasite-mediated balancing selection. Here, we examined diversity and differentiation of neutral microsatellite loci and functional MHC class I genes in house sparrows (*Passer domesticus*), living in six insular and six mainland populations, and we aimed to determine whether their diversity or differentiation correlates with the diversity and the prevalence of infection of hemosporidian parasites. We found that island bird populations tended to have lower neutral genetic variability, whereas MHC variability gene was similar between island and mainland populations. Similarly, island populations tended to show greater genetic differentiation than mainland populations, especially at microsatellite markers. The maintenance of MHC genetic diversity and its less marked structure in the island populations could be attributed to balancing-selection. The greater MHC differentiation among populations was negatively correlated with similarity in blood parasites (prevalence and diversity of parasite strains) between populations. Even at low prevalence and small geographical scale, haemosporidian parasites might contribute to structure the variability of immune genes among populations of hosts.

## Introduction

Habitat fragmentation is one of the greatest threats to the survival and persistence of wild populations (Morris and Doak 2002), and studies are needed to better understand how population fragmentation influences genetic diversity. Small and isolated populations have reduced genetic variability, increased inbreeding, and genetic drift that might increase their risk of extinction (Frankham, 1996, Frankham 1998; Luikart and Cornuet 1998; Keller and Waller 2002). Meta-analyses have indeed shown that species with low levels of genetic variability are prone to extinction (Reed and Frankham 2003; Spielman et al. 2004b). Reduced genetic diversity is expected to make small populations more susceptible to pathogens and parasites (Altizer et al. 2003; Spielman et al. 2004a) and fluctuations in other environmental conditions (Allendorf and Luikart 2007).

Neutral genetic variability is influenced by demographic factors, such as genetic drift and gene flow. Adaptive genetic variability is influenced by both demographic factors and selective factors. Thus, to provide a complete picture of the evolutionary potential of populations, it is necessary to assess the variability of both neutral and selected genes (Hoffman et al. 2003; Sommer 2005). Genes of the major histocompatibility complex (MHC) are excellent candidates for the study of functional or adaptive genetic variability. These highly polymorphic genes encode molecules that bind peptide antigens and present them to T cells, initiating an immune response (Hedrick 1994). MHC genes shape the pattern of resistance/susceptibility to a wide variety of parasitic diseases (Wegner et al. 2003; Bonneaud et al. 2006; Tollenaere et al. 2008; Oliver et al. 2009), and therefore, the diversity of MHC genes is generally assumed to be under balancing-selection, driven by parasite-mediated selection (PMS) (Doherty and Zinkernagel 1975; Bernatchez and Landry 2003). Gene conversion, sexual selection, and maternal–fetal interactions may also play a role (Edward & Hedrick 1998; Martinsohn et al. 1999; Penn and Potts 1999). Several authors have attempted to understand how PMS could generate and maintain the tremendous variation of MHC genes usually observed (Plachy et al. 1992; Sommer 2005; Spurgin and Richardson 2010). Three nonexclusive hypotheses of PMS have been proposed: (1) overdominance (also called “heterozygous advantage” or more accurately “heterozygote superiority”, Doherty and Zinkernagel 1975; Hughes and Nei 1992; Hedrick 1998); (2) negative frequency-dependent selection (also called “rare allele advantage”, Clarke and Kirby 1966; Slade and McCallum 1992); and (3) fluctuating selection in space and time (Hill 1991; Hedrick 2002). Any, or all, of these mechanisms could be operating at the same time. Therefore, despite the considerable amount of work that has been devoted toward identifying the form of selection acting on MHC genes, disentangling these hypotheses has been elusive (Apanius et al. 1997; Woelfing et al. 2009; Spurgin and Richardson 2010; but see Ejsmond et al. 2014; for a theoretical work).

One promising approach to investigate selection on MHC genes involves contrasting population structure at MHC and neutral loci across multiple populations (Miller and Withler 1997; Miller et al. 2001; Bernatchez and Landry 2003; Piertney 2003; Aguilar and Garza 2006; Bryja et al. 2007; Ekblom et al. 2007; Spurgin and Richardson 2010). Under heterozygote advantage, within-population adaptive variability is predicted to be higher than neutral variability, resulting in a lower MHC population structure (Schierup et al. 2000). Under spatially fluctuating selection, stronger MHC population structure is expected, relative to neutral loci (Charlesworth et al. 1997). Some studies have reported stronger differentiation at MHC genes than for neutral markers (Miller et al. 2001; Charbonnel and Pemberton 2005; Bryja et al. 2007; Ekblom et al. 2007; Loiseau et al. 2009; Spurgin and Richardson 2010), whereas other studies provide evidence that differentiation at the MHC is weaker than that at neutral loci (Boyce et al. 1997; Schierup et al. 2000; Hedrick et al. 2001; Huang and Yu 2003; Strand et al. 2012). However, none of these studies demonstrate explicitly variations in pathogen communities across populations (but see Charbonnel and Pemberton 2005 and Tobler et al. 2014).

The comparison of neutral and selected genes might also prove useful in disentangling the relative importance of selection versus demography (drift and migration) in the erosion/maintenance of genetic diversity. This issue is a central question in evolutionary and conservation biology. Threatened species are usually comprised of small and isolated and genetically depauperate populations that have undergone a reduction in numbers. However, it is difficult to infer the mechanisms leading to low genetic diversity without a comparison with populations with different demographic trends (e.g*.,* Garrigan and Hedrick 2001; Qiu-Hong et al. 2006; but see Miller et al. 2010; Strand et al. 2012). Comparing populations of a nonthreatened species that differ in both selection regimes and demography might prove useful in teasing apart the mechanisms underlying the maintenance/erosion of genetic diversity. Insular and mainland populations would be particularly well suited for this kind of comparison. Insular populations usually have smaller population size and reduced gene flow than mainland populations, making them more prone to drift (Frankham 1997, 1998). However, offsetting this insular biogeography theory suggests that organisms living on islands are usually less exposed to the risk of infectious diseases simply because islands harbor fewer parasite species than the mainland (Maitland et al. 2000; Moro et al. 2003; Lenaghan et al. 2006; Nieberding et al. 2006). This difference also applies to predators and competitors, and therefore, selection pressures usually strongly differ between island and mainland populations.

In this study, we investigated MHC and microsatellite genetic variability and differentiation among 12 (six insular and six mainland) populations of house sparrow (*Passer domesticus*). We also quantified the selective regime of each population by assessing their haemosporidian parasite communities and the prevalence of haemosporidian infection (“avian malaria”). Avian malaria is a widespread vectorborne disease of wild birds, extensively studied during the last decades (Fallon et al. 2005; Valkiūnas 2005). While haemosporidian parasites that coevolved with birds are usually nonlethal, some studies have shown that they could be detrimental to host fitness-linked traits (Van Riper et al. 1986; Atkinson and Van Riper 1991; Atkinson 1999; Williams 2005; Palinauskas et al. 2008; Cellier-Holzem et al. 2010; Knowles et al. 2010).

The specific aims of this study were to determine (1) whether island populations have low microsatellite and/or MHC genetic variability than mainland populations, (2) whether genetic differentiation among populations was different depending on the type of marker used (microsatellites or MHC), and (3) whether haemosporidian parasites could explain differences or similarities in microsatellite or MHC genetic differentiation.

## Material and Methods

### Sampling

**Figure 1:**
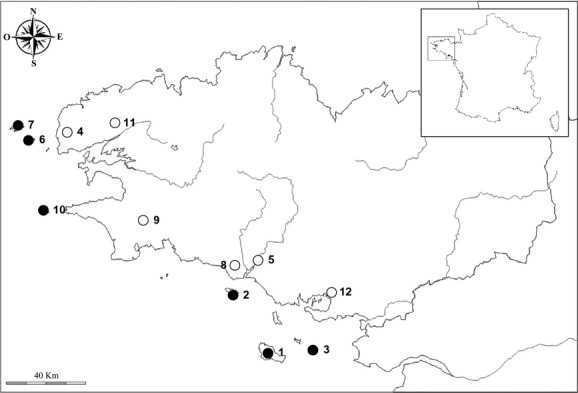
Map of Brittany, France, showing the geographical localization of 12 house sparrow populations used in this study. 1 – Belle-île, 2 – Groix, 3 – Hoedic, 4 – Kerinou, 5 – Languidic, 6 – Molène, 7 – Ouessant, 8 – Ploemeur, 9 – Quimper, 10 – Sein, 11 – St Elven, 12 – Vannes. Black dots localize island populations, and white dots localize mainland populations.

We sampled 12 populations of house sparrows located in Brittany, France (Fig.1). Four hundred and fifty adult house sparrows were captured between 2007 and 2009 using mist nets (Table1). Sample sizes varied between the different analyses as not all birds were successfully genotyped for the microsatellites, the MHC, or screened for parasites. Each bird was banded and visually sexed (the house sparrow being a sexually dimorphic species). We collected a small volume of blood (ca. 20 *μ*L) by brachial vein puncture. Blood was stored in 500 *μ*L of Queen's Lysis Buffer (QLB, Seutin et al. 1991). Once in the laboratory, DNA was extracted using the Wizard® SV 96 Genomic DNA Purification Kit (Promega, Wisconsin) according to the manufacturer's instructions, for subsequent molecular analyses.

**Table 1:** Sample characteristics, sample sizes, and genetic and parasite characteristics for each population studied

No	Population	Type	Years	Seasons	Microsatellites			MHC class I			Avian malaria parasites								
					*N*	*H*_E_	*A*	*N*	R	MHC/ind	*N*	Prevalence (%)	Shannon index	Number of different strains	SGS1	GRW11	TURDUS1	BLUTI02	SFC6
1	Belle-île	Island	2008	Autumn, winter	37	0.73	6.27	34	14.5	3	37	2.7	0	1	1	0	0	0	0
2	Groix	Island	2008	Autumn, winter	45	0.75	6.2	41	16.1	2.83	45	0	0	0	0	0	0	0	0
3	Hoedic	Island	2007, 2008	Autumn, winter	40	0.74	6.51	39	18.8	3.1	40	7.5	0.64	2	2	1	0	0	0
4	Kerinou	Mainland	2008	Spring	26	0.77	6.97	20	14	2.25	26	3.85	0	1	1	0	0	0	0
5	Languidic	Mainland	2008	Autumn, winter	55	0.73	6.49	53	17.9	3.23	55	23.64	0.79	4	10	1	1	0	1
6	Molène	Island	2008, 2009	Spring, winter	30	0.7	5.9	20	15	2.93	30	0	0	0	0	0	0	0	0
7	Ouessant	Island	2007, 2009	Spring, winter	71	0.71	6.41	60	11.8	2.25	69	8.45	0.71	2	0	0	2	3	1
8	Ploemeur	Mainland	2008	Winter	19	0.72	6.23	11	14.5	3.3	19	21.05	0.56	2	3	0	1	0	0
9	Quimper	Mainland	2008, 2009	Spring, winter	36	0.75	6.61	31	15	3	35	5.56	0	1	2	0	0	0	0
10	Sein	Island	2009	Winter	32	0.75	6.01	30	11.2	2.35	32	0	0	0	0	0	0	0	0
11	St Elven	Mainland	2008, 2009	Spring, winter	17	0.77	6.88	14	24.6	4.01	17	0	0	0	0	0	0	0	0
12	Vannes	Mainland	2008	Autumn, winter	41	0.75	6.69	36	14.2	2.89	41	24.39	0.67	2	6	4	0	0	0

*H*_E_ and *A* are, respectively, expected heterozygosity and allelic richness estimated for 11 individuals, obtained for microsatellite loci. *R* is MHC allelic richness estimated for 11 individuals. MHC/ind represents the mean number of MHC alleles per individual. SGS1, GRW11, TURDUS1, BLUTI02, and SFC6 are the name of the different parasite strains found in our populations. *N* gives the samples sizes for each marker and for avian malaria parasite assessment in each population.

### Microsatellite genotyping

A total of 449 individuals were genotyped using twelve microsatellite loci: PdomD09, PdomC11, PdomA08, PdomF09, PdomB01, PdomE09, PdomA04, PdomH05 (Garnier et al. 2009), Mjg1 (Li et al. 1997), Ase18 (Richardson et al. 2000), Pdo3, and Pdo5 (Griffith et al. 1999). Amplifications were carried out in a final volume of 10 *μ*L including 10–50 ng of DNA, 2 *μ*L of 5× buffer, 1.5–2 mmol/L of MgCl_2_, 400 *μ*L of dNTPs, 1 *μ*mol/L of each primer, and 0.2 U of *Taq* DNA polymerase (Promega). The theremocycling was performed using the following program: 94 °C 3 min, 30 cycles of 94 °C 20s, 20s for annealing (48 °C to 56 °C according to the locus), and 72 °C 40s, followed by a final extension of 72 °C 5 min. PCR amplifications were performed using fluorescent-labeled primers (6′). We used four different fluorochromes: PET for PdomD09, PdomB01, and Mjg1; FAM for PdomC11, PdomE09, and Ase18; VIC for PdomA08, PdomA04, and Pdo3; and NED for PdomF09, PdomH05, and Pdo5. Each locus was amplified separately, but eletrophoresed through an ABI3730 automated sequencer by combining in each run four differently colored loci. Allele sizes were determined using GeneMapper 3.0 software (Applied Biosystems Carlsbad, CA).

### MHC class I genotyping

A total of 389 house sparrows were genotyped by amplifying the major part of the exon 3 (208 bp compared to 268 bp for the entire exon) of the class I locus, which corresponds to the peptide-binding region (PBR) (Bonneaud et al. 2004). PCR amplifications were performed using a fluorescent (6′FAM)-labeled primer (A23M – GCG CTC CAG CTC CTT CTG CCC ATA) and an unlabeled primer (A21M – GTA CAG CGC CTT GTT GGC TGT GA) (Bonneaud et al. 2004). Amplifications were performed in a final volume of 10 *μ*L, which included 50–100 ng of genomic DNA, 0.5 *μ*L of each primer, and 5 *μ*L of *Taq* PCR Master Mix Kit (QIAGEN, Venlo, Netherlands) containing DNA polymerase, buffer, and dNTPs. The PCR program began with 5-min initial denaturing at 95 °C followed by 35 cycles of 30-sec denaturation at 94 °C, 90-sec annealing at 56 °C, and 90-sec extension at 72 °C. A final elongation step was run for 10 min at 72 °C. To control for PCR contaminations, we used two negative controls (for PCR and for electrophoresis).

MHC diversity was screened using capillary electrophoresis single conformation polymorphism (CE-SSCP) (described in Schaschl et al. 2008 and in Griggio et al. 2011). The fluorescent-labeled PCR samples were prepared for electrophoresis by combining 1 *μ*L PCR product with 8.75 *μ*L Hi-Di formamide and 0.25 *μ*L of in-house-prepared ROX size standard (DeWoody et al. 2004). This mix was heated for 5 min at 95 °C to separate the complementary DNA strands. Analyses were conducted in an automated DNA sequencer (ABI PRISM 3130 xl automated DNA Sequencer; Applied Biosystems, Carlsbad, CA). The retention time of allelic variants was assessed relative to the ROX size standard. GeneMapper v4.0 software was used to analyze the SSCP data.

### Parasite screening

A total of 446 individuals were screened for the presence of haemosporidian parasites (genus *Plasmodium* and *Haemoproteus*) with a nested PCR. This reaction amplified a 524-bp-long fragment of the cytochrome *b* gene of the two parasite genera (Waldenstrom et al. 2004). The primers used, HEAMF (5′-ATGGTGCTTTCGATATATGCATG-3′) and HAEMR2 (5′-GCATTATCTGGATGTGATAATGGT-3′), were defined in Bensch et al. 2000. This method is highly repeatable, with a lower limit of detection of one infected blood cell per 100,000. Samples from negative individuals were analyzed twice to avoid false negatives. We identified the parasite strain by sequencing PCR products and comparing these sequences to those on GenBank using the algorithm BLASTN (http://blast.ncbi.nlm.nih.gov/Blast.cgi). We also created alignments of our sequences and listed them in the public database MalAvi (Bensch et al. 2009).

### Analyses

#### Genetic variability

Among our 12 populations, five were sampled in two different years (Ouessant: 2007 [*n* = 59] and 2009 [*n* = 12]; Hoedic: 2007 [*n* = 14] and 2008 [*n* = 26]; Molène: 2008 [*n* = 5] and 2009 [*n* = 25]; Quimper: 2008 [*n* = 31] and 2009 [*n* = 5]; and St Elven: 2008 [*n* = 13] and 2009 [*n* = 4]). As we found no genetic differentiation at microsatellite loci between the 2 years for any population, we decided to pool the data for all years.

We tested each population–locus combination for deviations from Hardy–Weinberg equilibrium and for linkage disequilibrium between pairs of microsatellite loci, using exact tests implemented in GenePop 4.0 (Rousset 2008). Global deviation from Hardy–Weinberg equilibrium was investigated for each population using Fisher's exact tests. The assumption of neutrality of microsatellite loci was tested using the method implemented by Beaumont and Nichols (1996) in LOSITAN (Antao et al. 2008). Within-population genetic diversity of microsatellites was assessed by computing the expected heterozygosity (*H*_E_) using the software Genetix (Belkhir et al. 2004). Microsatellite allelic richness (*A*) was computed using a rarefaction index for the smallest sample size for one locus in one population (17 individuals in St Elven) using F_STAT_ 2.9.3 (Goudet 1995). *H*_E_ and allelic richness for microsatellites were compared between insular and mainland populations with Mann–Whitney tests.

MHC allelic frequencies were calculated using Arlequin 3.5 (Excoffier and Lischer 2010) as the number of individuals carrying a certain allele divided by the total allele count observed in the population. Total allele count is defined as the sum of alleles found per individual in a population (Loiseau et al. 2009). We note that this method of determining allele frequencies may underestimate the frequency of common alleles and overestimate the frequency of rare alleles (Ekblom et al. 2007). Genetic diversity for MHC was calculated by the number of different alleles found in each population and was estimated using richness cumulative curves (index of allelic richness, *R*) with the software ESTIMATES 7.5 (Colwell 2006) based on the smallest number of individuals genotyped (11 individuals in Ploemeur). We also calculated the mean number of MHC alleles per individual (MHC/ind). MHC allelic richness and MHC/ind were compared between insular and mainland populations with Mann–Whitney tests.

#### Population differentiation

Genetic differentiation between populations for microsatellite markers was measured using *F*_ST_ and tested for each pair of populations with exact tests implemented in GenePop 4.0 (Rousset 2008). Genetic differentiation between populations based on MHC class I genes was assessed using the software Arlequin 3.5 (Excoffier and Lischer 2010). We identified 95% confidence intervals of overall pairwise *F*_ST_ for microsatellites by bootstrapping over loci in F_STAT_ 2.9.3 (Goudet 1995). We assessed how many MHC pairwise *F*_ST_ confidence limits overlapped and tested the significance of this distribution with a chi-square test. Although *F*_ST_ is arguably the most reported statistic in population genetics, its use is still debated (Hedrick 2005; Jost 2008; Meirmans and Hedrick 2011; Whitlock 2011), so we also calculated *D* values (*D*_EST_, Jost 2008) for both neutral and adaptive markers. We calculated *D*_EST_ using the online program SMOGD v.1.2.5 (1000 bootstraps, Crawford 2010) and for MHC genes using SPADE (Chao and Shen 2010) (10.000 bootstraps). The pairwise *F*_ST_ and *D*_EST_ values for both markers were highly significantly correlated (Mantel tests; respectively *r* = 0.98, *P* < 0.0001 for microsatellites and *r* = 0.99, *P* < 0.0001 for MHC). We therefore report only *F*_ST_ in the rest of the manuscript, but supply *D*_EST_ values in Table S2.

For each marker type, we tested for isolation by distance using a Mantel regression of *F*_ST_/(1-*F*_ST_) against the natural logarithm of the geographical distance between populations (Rousset 1997), with statistical significance inferred in XLSTAT. A Mantel test was also used to characterize the relationship between pairwise microsatellite and MHC population differentiation (*F*_ST_). We also used a Mantel correlation to test whether the sea represented a barrier to gene flow (migration), as reduced gene flow would be expected to promote island–mainland population differentiation. We constructed a triangular matrix describing the presence (1)/absence (0) of water (the sea) between all population pairs and regressed it against the matrices of pairwise microsatellites and MHC *F*_ST_ values.

#### Parasite analyses

For each sparrow population, we assessed the number of different avian malaria strains that were present and the prevalence of infection (proportion of infected birds). The number of strains was compared between insular and mainland populations with Mann–Whitney tests. Differences in prevalence (binomial distribution) between insular and mainland populations were investigated using a general linear mixed model (GLMM) with population type (island and mainland), year, season, and sex as fixed factors, and population within type as a random factor. Parasite similarity was also correlated with the MHC *F*_ST_ matrix, while controlling for microsatellite differentiation, using a partial Mantel test.

To test the idea that genetic differentiation at the MHC depends on the dissimilarity between the parasite communities, we computed the Steinhaus similarity coefficient, *S* = 2*W*/(*A *+ *B*), where W is the sum of the minimal number of infected hosts for each parasite strain between two populations, and *A* and *B* are the sum of infected hosts in populations *A* and *B*, respectively. In four populations, none of the sampled birds were infected. In these cases, the similarity index of pairs of populations with zero prevalence was set to 1 because we considered that the absence of the parasites homogenizes the selection pressures exerted by these specific parasites. The matrix of parasite similarity was then correlated with the matrices of *F*_ST_ based on both microsatellites and MHC, again using Mantel tests.

Unless otherwise attested, statistical tests were performed in SAS 9.2 (SAS, 2011) and JMP 5.0 (SAS, 2002).

## Results

### Genetic variability

The number of microsatellite alleles varied from 5 to 26 and 42/769 exact tests (between all pairs of loci, in each population) showed a significant linkage disequilibrium at the 0.05 level, but not after sequential Bonferroni correction. Eight of 144 population–locus tests showed a significant deviation from Hardy–Weinberg equilibrium, but again, none remained so after sequential Bonferroni correction. None of the 12 Hardy–Weinberg equilibrium global tests were significant. We found no other evidence that our 12 microsatellite markers were under selection (Fig. S1, supplementary material). There was no difference in expected heterozygosity (*H*_E_) between island (median = 0.74) and mainland (median = 0.75) populations (Table1, *W*_1_ = 26.5, *P* = 0.19); however, allelic richness was lower in insular (median = 6.24) than that in mainland ones (median = 6.65) (*W*_1_ = 32, *P* = 0.02).

We observed 45 MHC alleles among our 12 populations. The maximal number of MHC alleles found in an individual was eight, suggesting that we amplified at least 4 MHC class I loci. Allelic richness (*R*) and mean number of MHC alleles per individual (MHC/ind) for each population are given in Table1. There were no differences in *R* (*W*_1_ = 21, *P* = 0.69) nor in MHC/ind (*W*_1_ = 26, *P* = 0.23) between island (medians = 14.75 and 2.88, respectively) and mainland populations (medians = 14.75 and 3.12, respectively).

### Population differentiation

Pairwise microsatellite *F*_ST_ values were moderate, varying between −0.0026 (between Vannes and Ploemeur) and 0.0445 (between Quimper and Molène) (Table2). A total of 89% (59/66) of pairwise *F*_ST_ values indicated significant population differentiation, and 66% (44/66) were still significant after sequential Bonferroni correction. All 15 *F*_ST_ values between island pairs were significant. A total of 75% of the 36 pairwise *F*_ST_ values between island and mainland populations and only 6.7% of the 15 *F*_ST_ values between mainland pairs were significant. We did not observe evidence for isolation by distance (Mantel test, *r* = 0.091, *P* = 0.47); however, there was a strong correlation between *F*_ST_ values and the presence/absence of sea between population pairs (Mantel test, *r* = 0.426, *P* < 0.0001).

**Table 2:** Pairwise *F*_ST_ by population pairs

Populations	Belle-île	Groix	Hoedic	Kerinou	Languidic	Molène	Ouessant	Ploemeur	Quimper	Sein	St Elven	Vannes
Belle-île	–	0.0127^*^^*^^*^	0.0179^*^^*^^*^	0.0088^*^^*^^*^	0.0082^*^^*^^*^	0.0417^*^^*^^*^	0.0165^*^^*^^*^	**0.0066**	0.0088^*^^*^^*^	0.0225^*^^*^^*^	**0.0109**	**0.0076**
Groix	0.01259^*^^*^^*^	–	0.0112^*^^*^^*^	0.0087^*^^*^^*^	0.0100^*^^*^^*^	0.0315^*^^*^^*^	0.0160^*^^*^^*^	0.0015^*^^*^^*^	0.0157^*^^*^^*^	0.0186^*^^*^^*^	**0.0029**	**0.0043**
Hoedic	0.01487^*^^*^^*^	0.00146	–	0.0127^*^^*^^*^	0.0108^*^^*^^*^	0.0337^*^^*^^*^	0.0148^*^^*^^*^	**0.0078**	0.0086^*^^*^^*^	0.0164^*^^*^^*^	0.0112^*^^*^^*^	0.0065^*^^*^^*^
Kerinou	−0.00405	0.00573	**0.01033**	–	**0.0079**	0.0327^*^^*^^*^	0.0123^*^^*^^*^	0.0060	0.0078^*^^*^^*^	0.0103^*^^*^^*^	0.0035	**0.0034**
Languidic	**0.00768**	0.00476	0.00916^*^^*^^*^	−0.00454	–	0.0313^*^^*^^*^	0.0159^*^^*^^*^	0.0064	**0.0094**	0.0238^*^^*^^*^	**0.0116**	**0.0050**
Molène	0.03891^*^^*^^*^	**0.01637**	0.00675	0.04454^*^^*^^*^	0.03416^*^^*^^*^	–	0.0322^*^^*^^*^	0.0292^*^^*^^*^	0.0445^*^^*^^*^	0.0362^*^^*^^*^	0.0372^*^^*^^*^	0.0328^*^^*^^*^
Ouessant	0.01171^*^^*^^*^	0.0012	0.00134	0.00785	0.00466	**0.0139**	–	0.0046	0.0165^*^^*^^*^	0.0211^*^^*^^*^	**0.0123**	0.0098^*^^*^^*^
Ploemeur	−0.00114	0.00268	0.00258	−0.01093	−0.00323	**0.02403**	0.00342	–	**0.0101**	0.0199^*^^*^^*^	0.0065	−0.0026
Quimper	**0.0106**	0.00168	0.00704	0.00288	−0.00031	**0.02302**	0.00267	0.00494	–	0.0199^*^^*^^*^	**0.0096**	**0.0052**
Sein	0.03925^*^^*^^*^	0.0235^*^^*^^*^	0.01542^*^^*^^*^	0.03994^*^^*^^*^	0.03899^*^^*^^*^	0.00212	0.023^*^^*^^*^	**0.02063**	0.03688^*^^*^^*^	–	0.0094^*^^*^^*^	0.0148^*^^*^^*^
St Elven	0.00837	0.00098	−0.00336	0.00703	0.0076	**0.01247**	−0.0001	0.00417	0.00843	**0.01512**	–	0.0050
Vannes	0.01369^*^^*^^*^	**0.00624**	0.00551	0.00354	−0.00122	0.02438^*^^*^^*^	0.00272	0.00036	0.0025	0.03215^*^^*^^*^	0.00641	–

The half-matrix on the top gives the *F*_ST_ estimated with microsatellites loci. The half-matrix on the bottom gives the *F*_ST_ estimated with MHC class I genes. *F*_ST_ values in bold represent significant differentiation tests. *F*_ST_ values followed by three stars represent differentiation tests still significant after sequential Bonferroni correction.

Pairwise *F*_ST_ values for MHC varied between −0.0109 (Ploemeur and Kerinou) and 0.0399 (Molène and Kerinou) (Table2). Twenty-eight of the 66 *F*_ST_ values were statistically significant, and 17 were still significant after sequential Bonferroni correction (Table2). A total of 53.3% of the 15 differentiation tests between island pairs were significant, 25% of the 36 tests between island and mainland populations, and 0% of the 15 tests between mainland pairs. *F*_ST_ values for MHC were not correlated with geographic distance (Mantel test, *r* = 0.008, *P* = 0.97), but they were strongly correlated with the presence/absence of sea between populations (Mantel test, *r* = 0.379, *P* = 0.0003).

**Figure 2:**
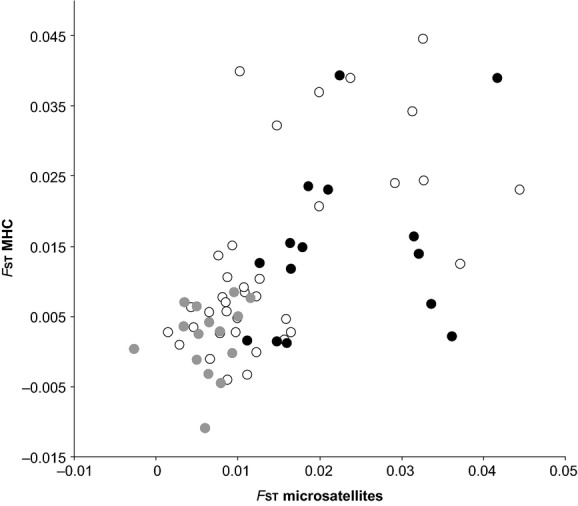
Correlation between microsatellite *F*_ST_ values and MHC genes *F*_ST_ values between all populations pairs. White circles represent all population pairs; black circles, island population pairs; and gray circles, mainland population pairs. Black line gives the linear regression between microsatellite *F*_ST_ values and MHC genes *F*_ST_ values for all population pairs.

The correlation between microsatellite *F*_ST_ and MHC *F*_ST_ was highly significant (Mantel test, *r* = 0.59, *P* < 0.0001, Fig.2), even after controlling for conditional variation due to the presence/absence of sea (*r* = 0.51, *P* < 0.0001). We found 7 pairwise comparisons where MHC-based *F*_ST_ were above the upper limit of the 95% CI of microsatellite-based *F*_ST_, 38 pairwise comparisons where MHC-based *F*_ST_ were within the 95% CI of microsatellite-based *F*_ST_, and 21 pairwise comparisons where MHC-based *F*_ST_ were below the lower limit of the 95% CI of microsatellite-based *F*_ST_. We found a significant excess of MHC-based *F*_ST_ inferior to microsatellite-based *F*_ST_ (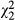 = 21.91, *P* < 0.0001).

### Avian malaria parasites

Among the 446 birds screened for haemosporidian *Plasmodium* and *Haemoproteus*, 40 individuals harbored the infection with five different parasite strains. Prevalence in each population is given in Table1. No parasites were found in 4 of the 12 populations (Groix, Molène, Sein, and St Elven). Prevalence did not vary between years (*F*_2,244_ = 3.01, *P* = 0.096), or sex (*F*_1,445_ = 1.03, *P* = 0.31). Prevalence was higher in winter (12.94%) than that in autumn (3.54%) and spring (3.85%) (*F*_2,244_ = 10.19, *P* = 0.002), and was higher in mainland (15.46%) than that in insular populations (3.92%) (*F*_1,445_ = 12.51, *P* = 0.0054).

**Figure 3:**
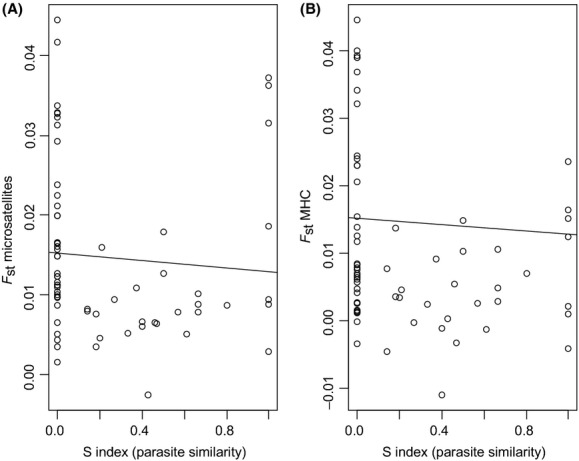
Plot between pairwise *F*_ST_ for microsatellites (A) and for MHC (B) and parasite similarity (Steinhaus index, S) between populations. The black line represents the linear regression.

We computed a Steinhaus similarity coefficient between pairs of populations (Table3) to putatively infer the similarity of selection pressures exerted by avian malaria on their local hosts. This parasite similarity matrix was not correlated with geographical distance (Mantel test, *r* = −0.141, *P* = 0.25), nor with the presence/absence of sea between two populations (Mantel test, *r* = −0.052, *P* = 0.67). There was no correlation between microsatellite *F*_ST_ and parasite similarity between populations (Mantel test, *r* = −0.076, *P* = 0.56, Fig.3). However, there was a negative correlation between MHC *F*_ST_ and parasite similarity (Mantel test, *r* = −0.246, *P* = 0.039, Fig.3), which remained significant after conditional when microsatellite variation was subtracted (partial Mantel test, *r* = −0.250, *P* = 0.032).

**Table 3:** Pairwise Steinhaus coefficient between all population pairs

Populations	Belle-île	Groix	Hoedic	Kerinou	Languidic	Molène	Ouessant	Ploemeur	Quimper	Sein	St Elven
Groix	0										
Hoedic	0.5	0									
Kerinou	1	0	0.5								
Languidic	0.14	0	0.38	0.14							
Molène	0	**1**	0	0	0						
Ouessant	0	0	0	0	0.21	0					
Ploemeur	0.4	0	0.57	0.4	0.47	0	0.2				
Quimper	0.67	0	0.8	0.67	0.27	0	0	0.67			
Sein	0	**1**	0	0	0	**1**	0	0	0		
St Elven	0	**1**	0	0	0	**1**	0	0	0	**1**	
Vannes	0.18	0	0.46	0.18	0.61	0	0	0.43	0.33	0	0

We attributed the coefficient 1 when the two populations had no malaria parasites. Values 1 in bold were excluded in the analyses excluding the comparisons between two populations without malaria parasites.

## Discussion

We found that (1) genetic variability of neutral genetic markers was lower in island compared to mainland populations, as expected by genetic drift, whereas variability at the class I MHC gene was similar between island and mainland populations. Similarly, island populations appear to be more differentiated than mainland populations. We also found (2) stronger genetic differentiation between bird populations at microsatellite markers than for MHC genes, which could indicate that balancing selection overrides divergent selection in this class of MHC gene. Finally, we found that (3) similarity in the composition (strains and prevalence of infection) of haemosporidian parasite community was correlated with the population differentiation based on MHC, even when controlling for conditional microsatellite-based differentiation. Below, we discuss each of these three main findings in more detail.

(1) Microsatellite allelic richness was lower in islands than that in mainland populations. This is a rather common pattern that has been reported in several island/mainland comparisons, likely due to smaller population sizes and reduced gene flow in insular populations. Frankham (1997) reviewed 202 studies where genetic diversity was compared between insular and mainland populations and showed that in 163 of such studies, insular populations had a rather lower diversity (decreased allelic richness though not heterozygosity). This finding might be due to the fact that allelic richness is more sensitive to recent decreases in population size than diversity indexes (such as heterozygosity, Luikart and Cornuet 1998). Unlike microsatellites, MHC genetic variability was similar between island and mainland populations, suggesting that selective forces might counteract the effect of drift on these genes even in small populations (Garrigan and Hedrick 2001; Gutierrez-Espeleta et al. 2001; Hedrick 2002; Aguilar et al. 2004).

Island populations were more differentiated than mainland populations at both genetic markers, suggesting a reduced gene flow between insular populations. The house sparrow is a sedentary species, with limited dispersal ability. Using recoveries, Paradis et al. (1998) estimated a mean dispersal distance of 1.7 km for juveniles and 1.9 km for adults. Similarly, Altwegg et al. (2000) found that only 10% of house sparrows were able to disperse between Norwegian islands, separated from 2 to 20 km. The insular and mainland populations used in our study are located within a relatively small area and the geographical distance between insular–mainland populations was similar to the distance between insular–insular and mainland–mainland pairs of populations. This relationship allowed us to disentangle the role of distance and a potential barrier to dispersal: the presence of sea. In agreement with the idea that the sea represents a major obstacle to house sparrow dispersal, we found that insular–insular and insular–mainland populations were more strongly differentiated regardless of the distance separating them and regardless of genetic marker.

(2) We found a stronger genetic differentiation between bird populations at microsatellite markers than for MHC genes. Disentangling the forces that drive the maintenance of variability in functionally important genes is challenging. Previous work conducted on nonmodel species has mostly aimed at comparing patterns of population differentiation based on neutral markers (supposedly reflecting only demographic forces) and selected genes (reflecting both neutral and selective forces). Such comparisons have provided useful insights on the selection acting on MHC. However, it should be kept in mind that direct comparisons of *F*_ST_ computed on different genetic markers are somewhat problematic if these markers differ in their rate of molecular evolution and their diversity, which is the case for microsatellites and MHC genes. The use of a standardized genetic differentiation estimator (Hedrick 2005) that controls for marker variability would counteract this problem. But, this measure needs a locus-specific approach, which is not yet the case for MHC genes, in nonmodel vertebrates. We also calculated, for microsatellites and for MHC genes, the Jost's *D* (*D*_EST_) (Jost 2008), which is a measure of genetic distance. *D*_EST_ appears to be a good indicator of differentiation, especially when mutation rate is high, relative to migration rate, which could be the case in insular populations.

Overall, differentiation based on MHC was weaker than for microsatellites. Weaker differentiation at MHC genes is in agreement with high overall MHC diversity relative to neutral diversity and with the idea that similar parasite pressures (under heterozygote or rare allele advantage) tend to homogenize allele frequencies and reduce population differentiation (Boyce et al. 1997; Schierup et al. 2000; Hedrick et al. 2001; Bernatchez and Landry 2003; Huang and Yu 2003).

It is worth stressing that the 12 populations studied here are located within a limited geographical scale of a few hundred kilometers. In previous work, Loiseau et al. (2009) compared 13 populations of house sparrow over a much larger geographical scale (mostly mainland populations). They found that genetic differentiation increased with distance (whereas in this study, we did not find any evidence of isolation by distance whatever the marker used). Loiseau et al. (2009) also found that genetic differentiation was stronger for MHC genes when pairs of populations were geographically distant, whereas for populations located at short distance, differentiation was stronger for microsatellite markers. The results reported in the present study seem therefore to corroborate those findings, as at our small geographical scale, balancing selection appears to homogenize MHC allele frequencies.

(3) Finally, we found that similarity in the composition of haemosporidian parasite community was correlated with the population differentiation based on MHC. Identifying the selective agents responsible for the observed pattern is obviously not easy. Previous work has suggested that haemosporidian parasites might play a role in the maintenance of among population diversity of MHC genes (Jarvi et al. 2004; Bonneaud et al. 2006; Loiseau et al. 2011; and Westerdahl 2007 for a review in passerine birds). Island/mainland comparisons can be a powerful way to infer the role of parasites in the maintenance of MHC diversity. Insular populations are usually exposed to a lower diversity of pathogens, but they are usually more susceptible to the devastating effect of newly incoming pathogens *(*e.g*.,* Maitland et al. 2000; Moro et al. 2003; Nieberding et al. 2006 Barrientos et al. 2014).

We investigated the avian malarial community infecting each of the studied populations, and we found, as expected, that the prevalence of infection was lower in insular populations compared to mainland (even though, it should be noted that prevalence was generally low, certainly reflecting local environmental features, Loiseau et al. 2013). However, parasite strain diversity was similar between population types (islands and mainland), showing that mainland populations are not exposed to a wider spectrum of haemosporidian parasites. This finding probably arises because other bird species, with better dispersal skills than house sparrows, may distribute these parasite strains across insular and mainland populations. When combining strain diversity and prevalence in a single similarity index (Steinhaus similarity coefficient), we found that population differentiation based on MHC genes was stronger when the similarity in malaria parasites was weak. In other words, the stronger the dissimilarity in the composition of malaria parasites, the stronger the differentiation at MHC among host populations. This pattern was quite specific to the type of marker used, as using microsatellite-based *F*_ST_ did not produce the same results. Interestingly, these results suggest that even within a small geographical scale, with relatively homogeneous environmental characteristics, fine-tuned differences in parasite-exerted selection pressures can affect population differentiation of immune genes.

In conclusion, our findings suggest that despite the erosion of neutral genetic diversity of birds living on islands, balancing selection on the MHC genes seems to be strong enough to counteract genomewide genetic drift. Balancing selection also appears to explain the weaker MHC genetic differentiation than for microsatellites, in the house sparrow populations. Even at low prevalence and across a limited geographical scale, haemosporidian parasites may help drive the structure of MHC diversity between populations.

## Supporting Information

**Fig. S1** Graphical output with the simulated confidence area for neutral loci with *F*_ST_ of our 12 microsatellite loci.

**Table S1.** Pairwise *D*_EST_ by population pairs.
