# Supplementary material for: Genetic structure in insular and mainland populations of house sparrows (Passer domesticus) and their hemosporidian parasites
Source: Ecol Evol. 2015 Mar 23;5(8):1639–52. doi: 10.1002/ece3.1452 (PMC4409412; doi:10.1002/ece3.1452)
Supplement: Supplementary file 1 [file ece30005-1639-sd1.pdf]

Supporting information : Fig. S1

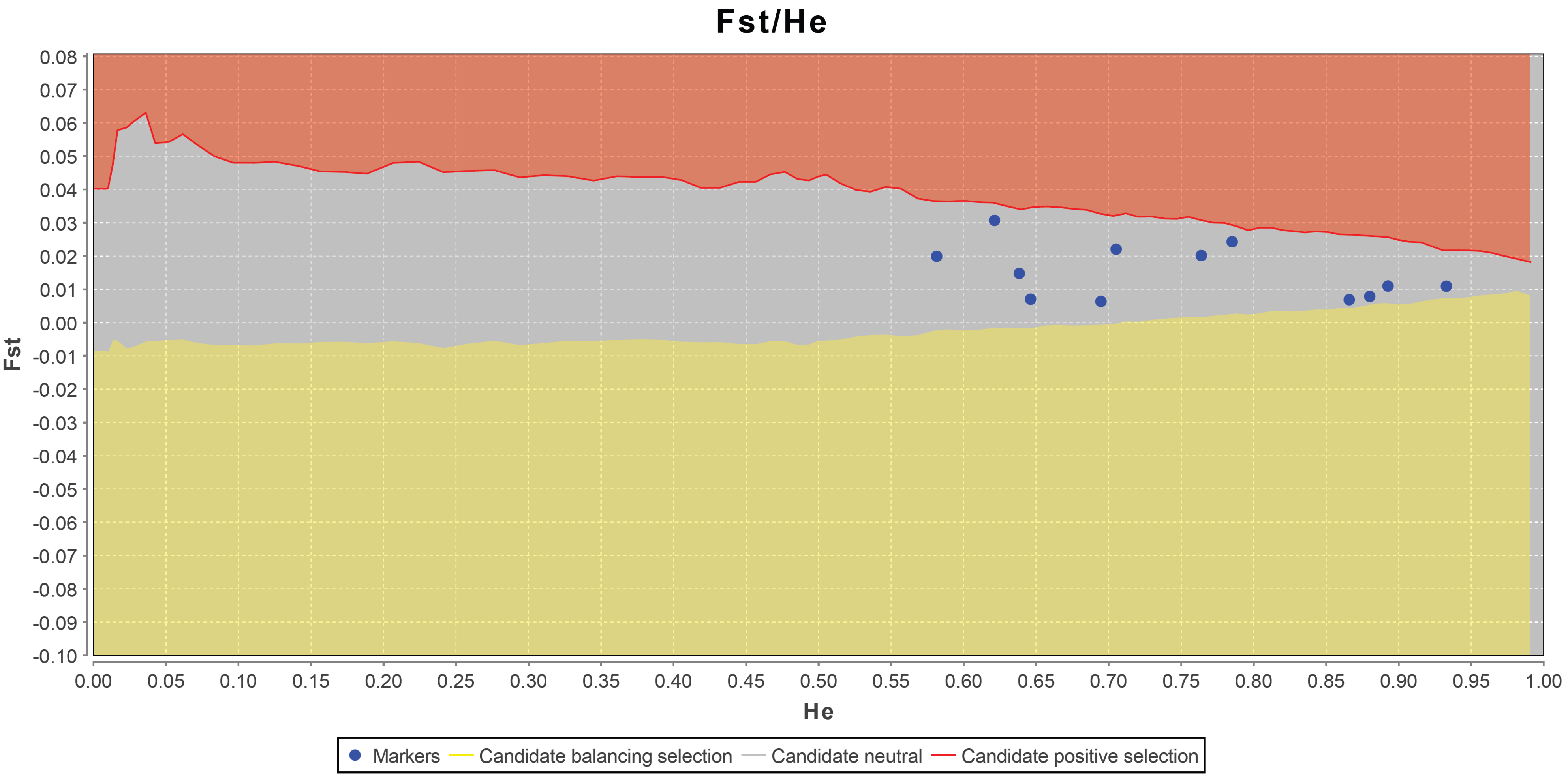

Fig. S1 Graphical output with the simulated confidence area for neutral loci (grey band) with F_{ST} of our 12 microsatellites loci represented as blue dots.

The red band represented F_{ST} area for positive selection, while the yellow band area for balancing selection.
