# Supplementary material for: Genetic structure in insular and mainland populations of house sparrows (Passer domesticus) and their hemosporidian parasites
Source: Ecol Evol. 2015 Mar 23;5(8):1639–52. doi: 10.1002/ece3.1452 (PMC4409412; doi:10.1002/ece3.1452)
Supplement: Supplementary file 2 [file ece30005-1639-sd2.pdf]

Supporting information : Table S1

Table S1 Pairwise D_{EST} by population pairs.

Populations	Belle-île	Groix	Hoedic	Kerinou	Languidic	Molène	Ouessant	Ploemeur	Quimper	Sein	Saint Elven	Vannes
Belle-île	-	0.01865	0.02692	0.01060	0.00794	0.07399	0.02505	0.00552	0.01749	0.03594	0.01267	0.00784
Groix	0.14900	-	0.01052	0.01250	0.01708	0.05336	0.02125	-1.8618044347e-08	0.03285	0.03458	-6.98146956171e-07	0.00174
Hoedic	0.19200	0.02400	-	0.01546	0.01899	0.06980	0.01415	0.00167	0.00965	0.03007	0.00141	0.00740
Kerinou	-0.03500	0.06600	0.12800	-	0.00541	0.05848	0.01664	0.00447	0.01400	0.01414	0.00142	0.00403
Languidic	0.07600	0.05700	0.12100	-0.04000	-	0.04809	0.01930	0.01154	0.00536	0.04837	0.00289	0.00937
Molène	0.48700	0.26500	0.12200	0.52300	0.44100	-	0.05773	0.03196	0.06766	0.06777	0.07114	0.06307
Ouessant	0.14700	0.01900	0.02400	0.09600	0.06000	0.24000	-	0.00085	0.01896	0.03084	0.00070	0.01037
Ploemeur	-0.01400	0.03800	0.04200	-0.10500	-0.03600	0.34200	0.05200	-	0.00994	0.01653	0.00054	5.48686467638e-05
Quimper	0.09900	0.02000	0.09000	0.02500	-0.00300	0.27700	0.03500	0.04800	-	0.05045	0.00861	0.00735
Sein	0.47000	0.35700	0.25800	0.44800	0.48200	0.03300	0.37100	0.27600	0.43200	-	0.00764	0.02480
Saint Elven	0.11000	0.01600	-0.06900	0.08900	0.10200	0.24500	-0.00400	0.06900	0.10900	0.27100	-	-4.56140680265e-06
Vannes	0.14900	0.08600	0.08300	0.03800	-0.01400	0.35600	0.04000	0.00500	0.02700	0.44600	0.10100	-

The half-matrix on the top gives the D_{EST} estimated with microsatellites loci. The half-matrix on the bottom gives the D_{EST} estimated with MHC class I genes.
